# Regarding: In Vivo Evaluation of Decellularised Skeletal Muscle Matrices for Skeletal Muscle Repair: A Systematic Review

**DOI:** 10.1111/jcmm.70888

**Published:** 2025-10-09

**Authors:** DuJiang Yang, Lin Yang, Shuang Wang, GuoYou Wang

**Affiliations:** ^1^ The Affiliated Traditional Chinese Medicine Hospital, Southwest Medical University Luzhou People's Republic of China; ^2^ Center for Orthopedic Diseases Research, the Affiliated Traditional Chinese Medicine Hospital, Southwest Medical University Luzhou People's Republic of China

Dear Editor,

1

We read with great interest the systematic review by Hennion et al. [1] which comprehensively evaluates the in vivo performance of decellularised skeletal muscle matrices (DSMMs) for muscle repair. Their work provides a valuable synthesis of current evidence regarding the regenerative potential of DSMMs in volumetric muscle loss treatment. While the authors thoroughly address key parameters influencing DSMM efficacy, several aspects warrant further discussion to advance this promising field.

The review appropriately highlights the critical importance of preserving native extracellular matrix (ECM) architecture to facilitate cellular infiltration and vascularisation [1]. However, the substantial heterogeneity in decellularisation protocols across studies—ranging from detergent‐based to enzymatic methods—deserves more emphasis regarding its impact on residual DNA content, glycosaminoglycan preservation and ultimate biocompatibility [2]. Recent advances suggest that establishing standardised metrics for decellularisation efficiency (e.g., ≤ 50 ng DNA/mg tissue) and mechanical integrity (e.g., Young's modulus matching native tissue) is crucial for meaningful cross‐study comparisons [3].

While the review discusses immune responses to DSMMs, the role of macrophage polarisation in long‐term outcomes requires deeper exploration. Emerging evidence indicates that ECM‐bound cytokines (e.g., TGF‐β, IL‐10) can be strategically leveraged to steer immune responses toward pro‐regenerative phenotypes [4]. Furthermore, the potential of DSMMs to modulate neuroimmunological pathways, particularly those involving aryl hydrocarbon receptor (AhR) signalling—a key regulator of inflammation and tissue repair—merits investigation given its emerging role in mitigating fibrosis in other regeneration models [5].

The comprehensive cataloguing of functional outcomes appropriately highlights the variability in assessment methods across studies. Integration of multimodal evaluation frameworks, including gait analysis for large animals and advanced ultrasonography for angiogenesis monitoring, could significantly enhance translational predictability [6]. The limited discussion of long‐term (> 12 weeks) data represents an important gap, as understanding fibrotic progression and ectopic mineralisation risks is essential for clinical adoption [7].

Regarding translational challenges, the review notes scalability issues but could expand on recent biomaterial innovations addressing these limitations. For instance, 3D‐bioprinted DSMM scaffolds with architecturally patterned microchannels have demonstrated enhanced myoblast alignment and neural integration in large animal models [8]. Similarly, while crosslinking strategies balance degradation kinetics with mechanical stability, their long‐term biocompatibility requires more thorough validation [9]. The integration of stimuli‐responsive materials, such as ultrasound‐activated components, shows promise for enabling non‐invasive monitoring and targeted therapeutic release in regenerative contexts [10].

In conclusion, Hennion et al. provide a valuable foundation for DSMM research. Future studies should prioritise: standardised decellularisation protocols, comprehensive long‐term immunological monitoring, and innovative biomaterial approaches to accelerate the translation of DSMMs from preclinical success to clinical solutions for muscle repair.

Sincerely,

DuJiang Yang, MD

Guoyou Wang, MD, PhD

President and Party Secretary of the Southwest Medical University Hospital of traditional Chinese medicine

## Author Contributions

**DuJiang Yang:** conceptualization, writing – original draft, methodology, writing – review and editing, data curation. **Lin Yang:** investigation, validation. **Shuang Wang:** investigation, validation. **GuoYou Wang:** funding acquisition, validation.

## Ethics Statement

The authors have nothing to report.

## Consent

The authors have nothing to report.

## Conflicts of Interest

The authors declare no conflicts of interest.

## Data Availability

Data sharing not applicable to this article as no datasets were generated or analysed during the current study.
